# RNAi of *HvMMP2* Affects Larval-Pupal Transition and Adult Eclosion in the *Henosepilachna vigintioctopunctata*

**DOI:** 10.3390/insects17050494

**Published:** 2026-05-13

**Authors:** Jian-Jian Wu, Meng-Yue Chang, Chen-Yi Wang, Yi-Fan Guo, Kun-Peng Cui, Hao Yu

**Affiliations:** Exotic Invasive Species Biosecurity Control Innovation Team, School of Plant Protection and Environment, Henan Institute of Science and Technology, Xinxiang 453003, China; achangyaa@163.com (M.-Y.C.); 17527055765@163.com (C.-Y.W.); 15333732803@163.com (Y.-F.G.); 15538963761@163.com (K.-P.C.)

**Keywords:** *Henosepilachna vigintioctopunctata*, matrix metalloproteinase 2, metamorphosis, RNAi

## Abstract

*Henosepilachna vigintioctopunctata* is a major pest of solanaceous crops. Matrix metalloproteinase 2 (*MMP2*) is a zinc ion-dependent endopeptidase that plays a crucial role in the remodeling process of the extracellular matrix (ECM) within cells. However, the function of *HvMMP2* in *H. vigintioctopunctata* remains unknown. In this study, we cloned and characterized the *HvMMP2* gene in *H. vigintioctopunctata* and investigated its function using RNA interference (RNAi). *HvMMP2* exists as two transcript variants that differ at the 5′ end. *HvMMP2* is highly expressed in the prepupal stage, the pupal stage and the intestine. Silencing *HvMMP2* expression in fourth-instar larvae led to approximately 54% mortality at the prepupal stage, with the remaining larvae dying after pupation. RNAi with *HvMMP2* in third-instar larvae did not affect their development to the fourth instar, but caused mortality in the majority of larvae during the prepupal and pupal stages, and most of these pupae exhibited wing deformities. Examination of these stunted larvae by dissection showed that their fat bodies were abnormally shaped and that yellow uric acid crystals had accumulated in the Malpighian tubules. Collectively, our findings indicate that *HvMMP2* plays a critical role in pupation and eclosion in *H. vigintioctopunctata* and support *HvMMP2* as a potential molecular target for further RNAi-based control studies.

## Simple Summary

The role of matrix metalloproteinases 2 (*MMP2*) in many non-model insect pests remains unclear. In this study, we demonstrated that ds*HvMMP2* treatment was associated with metamorphic defects and high mortality in *Henosepilachna vigintioctopunctata* larvae. Our findings support *HvMMP2* as a potential candidate target for RNAi-based control.

## 1. Introduction

*Henosepilachna vigintioctopunctata* (Coleoptera: Coccinellidae) is one of the most destructive insect pests in Asian countries, particularly in China, India, and Japan [1,2,3]. Outside Asia, this beetle is also found in Brazil and Australia [4]. It infests a wide range of solanaceous crops, including potato, eggplant, tomato, pepper, and tobacco, as well as cucurbits such as cucumber, white gourd, and loofah [5]. Both larval and adult stages cause considerable damage, resulting in crop yield losses of approximately 60% [6]. Due to the extensive and frequent use of chemical pesticides, several problems have been caused, such as environmental pollution, pest resistance, and crop quality degradation [7]. Therefore, the development of alternative strategies for sustainable plant protection is urgently needed.

In recent years, due to its high targeted specificity and environmental friendliness, RNAi has been developed as an environmentally friendly and less risky pest management strategy. The first RNAi-based pesticide, Ledprona, was approved for commercialization in the United States in 2023, primarily to control the Colorado potato beetle (https://www.epa.gov/pesticides/epa-registers-novel-pesticide-technology-potato-crops, accessed on 9 March 2025). However, currently, there is a lack of effective target gene resources for RNAi, and existing research mainly focuses on genes related to chitin synthesis and degradation in insect cuticles [8,9,10,11,12], energy metabolism [13,14,15], and hormone signaling pathways [6,16,17,18]. In addition, multiple factors can affect the efficiency of RNAi, among which the stability of dsRNA is a key determinant. Exposed dsRNA will not only be degraded by insect nucleases but also significantly reduce the field control effect due to ultraviolet rays in sunlight [19]. Although using nanoparticle complexes (such as chitosan, liposomes, star-shaped cationic polymers (SPCs), layered double hydroxides (LDH), and glycosylated polymers (GNP)) to encapsulate dsRNA can improve transduction efficiency and reduce cytotoxicity to promote the cellular uptake of dsRNA, and can also reduce the risk of nuclease degradation under environmental conditions (such as temperature changes and changes in culture media) [20,21], but the cost is high and it is not easy to be promoted. In conclusion, insecticides based on RNAi have great potential and will undoubtedly become a pillar of future pest management.

Matrix metalloproteinases (MMPs) are a conserved family of zinc-dependent endopeptidases that play pivotal roles in extracellular matrix (ECM) remodeling by degrading various ECM components, including collagens, proteoglycans, and glycoproteins [22,23,24]. In insects, MMPs have been shown to be essential for multiple developmental events, including embryogenesis, organ morphogenesis, and metamorphosis [24,25,26]. In *Drosophila melanogaster*, differential expression of *MMP2* controls growth anisotropy of the ECM envelope [27]. In the beetle *Tribolium castaneum*, MMPs regulate tracheal and gut development as well as innate immunity [24]. Moreover, in the silkworm *Bombyx mori*, *MMP2* has been implicated in ovarian development by degrading collagen I [28], and in the moth *Helicoverpa armigera*, *MMP2* promotes brain development during metamorphosis under the regulation of the steroid hormone 20-hydroxyecdysone [29]. These studies highlight the functional diversity and evolutionary conservation of MMPs in insect development.

Despite these advancements, the role of matrix metalloproteinases (MMPs) in many non-model insect pests remains unclear. In this study, we identified and characterized *HvMMP2*, a homolog of insect *MMP2*, in *H. vigintioctopunctata*. We investigated its expression patterns during larval development and assessed its biological functions using RNA interference, and assessed whether *HvMMP2* may represent a candidate target for RNAi-based control approaches.

## 2. Materials and Methods

### 2.1. Insect

The laboratory population of the 28-spotted ladybird beetle was established from individuals collected on eggplant leaves in the vegetable fields surrounding Henan Institute of Science and Technology in Xinxiang in June 2023. The beetles were reared in the laboratory under controlled conditions (temperature: 25 ± 1 °C, relative humidity: 70–80%, photoperiod: 14 L:10 D) using eggplant and potato leaves as food sources. Under these rearing conditions, the larvae developed through four distinct instars, with average durations of 3 days for the first instar, 2 days for the second instar, 2 days for the third instar, and 3 days for the fourth instar. Upon reaching full maturity, fourth-instar larvae ceased feeding, attached the posterior end of the abdomen to the substrate surface, and entered the prepupal stage. The prepupal stage lasted approximately 2 days, followed by a pupal stage of about 4 days, after which adults emerged.

### 2.2. Molecular Cloning

Two *HvMMP2* transcript variants were obtained from the transcriptome database of *H. vigintioctopunctata*. The correctness of these sequences was verified through polymerase chain reaction (PCR) using the primers listed in Table S1. The two sequenced cDNAs were submitted to GenBank (accession numbers: *HvMMP2x1*, PX842711; *HvMMP2x2*, PX842712).

The domains of *HvMMP2* transcript variants were predicted by NCBI Conserved Domain Search (https://www.ncbi.nlm.nih.gov/Structure/cdd/wrpsb.cgi?, accessed on 19 March 2024). The domains of *HvMMP2* transcript variants were compared with those derived from *Tribolium castaneum*, *Dendroctonus ponderosae* and *Bactrocera dorsalis* by software of GENEDOC (2.7, National Resource for Biomedical Supercomputing, NRBSC, Urbana, IL, USA). Phylogenetic analysis of *MMP2* sequences downloaded from NCBI (https://www.ncbi.nlm.nih.gov/, accessed on 19 March 2024) was constructed by using MEGA 6.0 (https://megasoftware.net/download_form, accessed on 19 March 2024) with the neighbor joining (NJ) method and the bootstrap value was set as 1000.

### 2.3. Preparation of dsRNAs

Due to the extremely short lengths of the different regions of the two transcriptional variants of *HvMMP2*, it is not possible to design corresponding dsRNA fragments (Figure S1). Therefore, we have designed dsRNA specifically for the common areas of both of them for research purposes. Using the E-RNAi website (https://dsrna-engineer.cn/, accessed on 19 March 2024), specific primers (ds*HvMMP2*-1 and ds*HvMMP2*-2) were designed for different regions of the conserved sequence of the *HvMMP2* gene, and double-stranded RNA was synthesized. The lengths of the two dsRNA fragments are 229 bp and 346 bp respectively. To prevent the risk of off-target effects, these targeted regions were further BLAST (BLASTN) (https://blast.ncbi.nlm.nih.gov/Blast.cgi?PROGRAM=blastn&PAGE_TYPE=BlastSearch&LINK_LOC=blasthome, accessed on 19 March 2024) searched against the *H. vigintioctopunctata* transcriptome to identify any possible off-target sequences that had an identical match of 20 bp or more. The T7 promoter sequence “taatacgactcactataggg” was added to the 5′ end of both the forward and reverse primers. The specific primers used to clone the fragments for ds*HvMMP2* synthesis are listed in Table S1.

Double-stranded RNA (dsRNA) was synthesized following the standard protocol of the T7 RNAi Transcription Kit (Vazyme, Nanjing, China). The dsRNA synthesis reaction mixture consisted of 2 μL of 10× Transcription Buffer, 8 μL of NTP Mix, 1 μg of PCR-purified product, and 2 μL of T7 Enzyme Mix, with the final volume adjusted to 20 μL using RNase-free water. The mixture was gently mixed and incubated at 37 °C for 16 h in a water bath, followed by cooling. To remove the DNA template, 1 μL of DNase I, 2 μL of RNase T1 (10 U/μL), and 17 μL of RNase-free water were added to the reaction mixture. After thorough mixing, the mixture was incubated at 37 °C for 30 min. Subsequently, 2 μL of sodium acetate solution and 50 μL of 95% ethanol were added, mixed well, and centrifuged at 12,000× *g* for 10 min at 4 °C. The resulting pellet was retained and washed twice with 1 mL of freshly prepared 70% ethanol. The pellet was air-dried at room temperature and then dissolved in 50 μL of RNase-free water. The yield of dsRNA was determined by measuring absorbance at 260 nm with a NanoDrop OneC spectrophotometer (Thermo Fisher Scientific, Waltham, MA, USA), and its integrity was verified by agarose gel electrophoresis. The dsRNA was stored at −80 °C in an ultra-low temperature freezer.

### 2.4. Microinjection for RNAi

The same method as before was used to inject dsRNA [17,30,31]. In simple terms, an equal volume (0.1 μL) of a solution containing 400 ng of dsRNA was injected into the newly hatched fourth-instar larvae, and 300 ng of dsRNA was injected into the newly hatched third-instar larvae. The negative control larvae were treated with an equal volume of ds*EGFP* solution. In order to enable the processed larvae to start feeding as soon as possible, prior to injection, larvae were starved for at least 2 h. Following injection, larvae were reared on fresh potato leaves until they reached the prepupal stage. Each treatment contained 12 larvae, with six biological replicates. For sampling, three replicates were collected 24 and 48 h post-injection for qRT-PCR analysis to assess RNAi efficiency. Another three replicates were maintained for a 3-week observation period to monitor defective phenotypes, pupation rate, and emergence rate.

### 2.5. Real-Time Quantitative PCR (qRT-PCR)

For temporal expression analysis, RNA templates were obtained from eggs (day 3), first- to fourth-instar larvae, prepupae, pupae, and adults. For tissue expression pattern analysis, RNA templates were derived from the foregut, midgut, hindgut, Malpighian tubules, epidermis, and fat body of day-2 final-instar larvae. Each sample contained 20–30 individuals and was repeated three times. To assess the effects of treatments, total RNA was extracted from treated larvae. Each sample contained 12 individuals and was repeated three times. Total RNA was extracted using the Trizol method, and first-strand cDNA was synthesized using the EasyScript All-in-One Reverse Transcription Kit (TransGen, Beijing, China). Quantitative mRNA measurements were performed by qRT-PCR in technical triplicate, using 2 internal control genes (*HvRPS18* and *HvRPL13*; the primers are listed in Table S1) according to the published results [32]. An RT negative control (without reverse transcriptase) and a non-template negative control were included for each primer set to confirm the absence of genomic DNA and to check for primer–dimer or contamination in the reactions, respectively.

According to a previously described method [33], the generation of specific PCR products was confirmed by gel electrophoresis. The primer pair for each gene was tested with a 5-fold logarithmic dilution of a cDNA mixture to generate a linear standard curve (crossing point (CP) plotted vs. log of template concentration), which was used to calculate the primer pair efficiency. All primer pairs amplified a single PCR product with the expected sizes, showed a slope of less than −3.0 and exhibited efficiency values ranging from 2.5 to 2.6. The standard curve of the quantitative primer q*HvMMP2* is y = −3.2164x + 32.292, with R^2^ = 0.9936. Data were analyzed by the 2^−ΔΔCT^ method, using the geometric mean of the two internal control genes for normalization. Set 3L1d, hindgut and ds*EGFP* treatment to 1, respectively.

### 2.6. Data Analysis

Statistical analyses were performed using SPSS Statistics 25 for Windows (Chicago, IL, USA). All figures were generated using GraphPad Prism v8.3 software and presented as the mean ± SE. Means were compared using Tukey’s test at *p* < 0.05. Variation between the control and treatment was tested using ANOVA (Breslow pairwise comparison, *p* < 0.05). The larva mortality rate is equal to the number of dead larvae/12, the pupation rate is equal to the number of pupae formed/12, the ratio of malformed pupae is equal to the number of malformed pupae/the number of pupae formed, and the emergence rate is equal to the number of emerged pupae/the number of pupae formed. In the experiment, no significant differences were observed between the dsRNAs targeting two different regions (ds*HvMMP2*-1 and ds*HvMMP2*-2) of the *HvMMP2* gene. Therefore, the data of this gene were combined. The individual results have been presented in Figure S2.

## 3. Results

### 3.1. Identification and Sequence Analysis of HvMMP2

**Figure 1:**
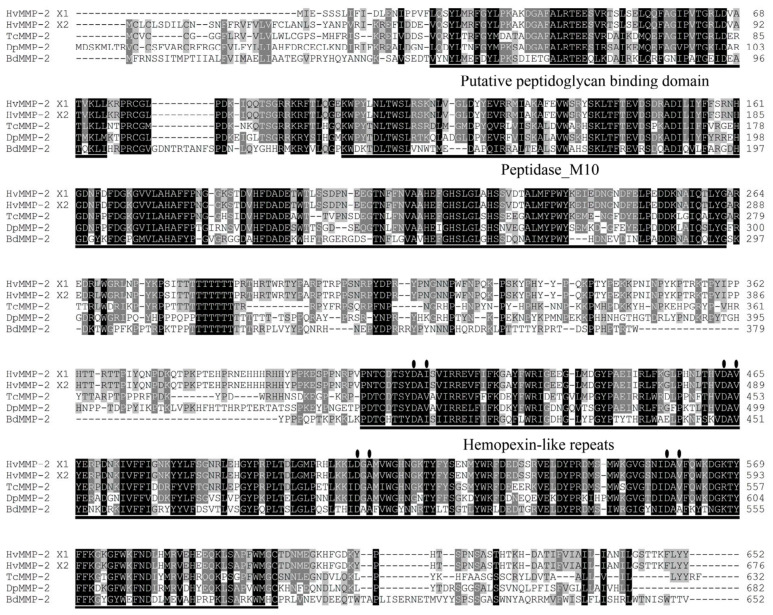
Amino acid multiple sequence alignment and analysis of conserved domains of *HvMMP2*. The amino acids of *MMP2* from *Henosepilachna vigintioctopunctata*, *Tribolium castaneum*, *Dendroctonus ponderosae* and *Bactrocera dorsalis* were subjected to multiple sequence alignment. Increasing background intensity (from light to dark) indicates an increase in sequence similarity. Gaps are introduced to permit alignment. Hemopexin-like repeats, putative peptidoglycan binding domains and Peptidase_M10 domains are respectively marked with black solid lines. The metal binding sites are marked with black ellipses.

**Figure 2:**
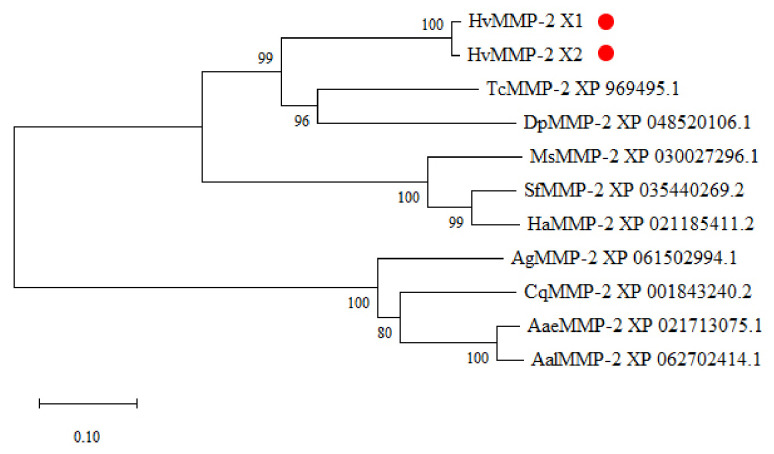
Evolutionary tree analysis of HvMMP2. The MMP2 sequences used for constructing the evolutionary tree were respectively derived from Coleoptera insects (*Henosepilachna vigintioctopunctata*, *Tribolium castaneum* and *Dendroctonus ponderosae*), Lepidoptera insects (*Manduca sexta*, *Spodoptera frugiperda* and *Helicoverpa armigera*), and Diptera insects (*Anopheles gambiae*, *Culex quinquefasciatus*, *Aedes aegypti* and *Aedes albopictus*). The tree is constructed using the neighbor-joining method based on the full-length protein sequence alignments. Bootstrap analyses of 1000 replications are carried out, and bootstrap values > 60% are shown on the tree. The position of HvMMP2 in the evolutionary tree is marked with red circles.

By mining the transcriptome data, two full-length *HvMMP2* cDNAs, namely *HvMMP2x1* and *HvMMP2x2*, were cloned from *H. vigintioctopunctata*. The full-length sequences of *HvMMP2x1* and *HvMMP2x2* are 1959 bp and 2031 bp, respectively. Both transcript variants share the majority of the open reading frame but possess isoform-specific 5′ untranslated regions (UTRs) (Figure S1). Correspondingly, the deduced proteins share identical core regions, with short isoform-specific amino acid sequences at the N-terminus (Figure 1). Both isoforms contain Peptidase_M10, hemopexin-like repeats, and putative peptidoglycan-binding domains. Notably, the Peptidase_M10 domain includes a zinc-binding motif (HEXXHXXGXXH), indicating that these are proteases that cleave polypeptides and require zinc for catalytic activity. Phylogenetic analysis revealed that *HvMMP2* forms a single clade with the *MMP2* orthologs of *Tribolium castaneum* and *Dendroctonus ponderosae* within the order Coleoptera, suggesting a close evolutionary relationship (Figure 2).

### 3.2. The Expression Profiles of HvMMP2

**Figure 3:**
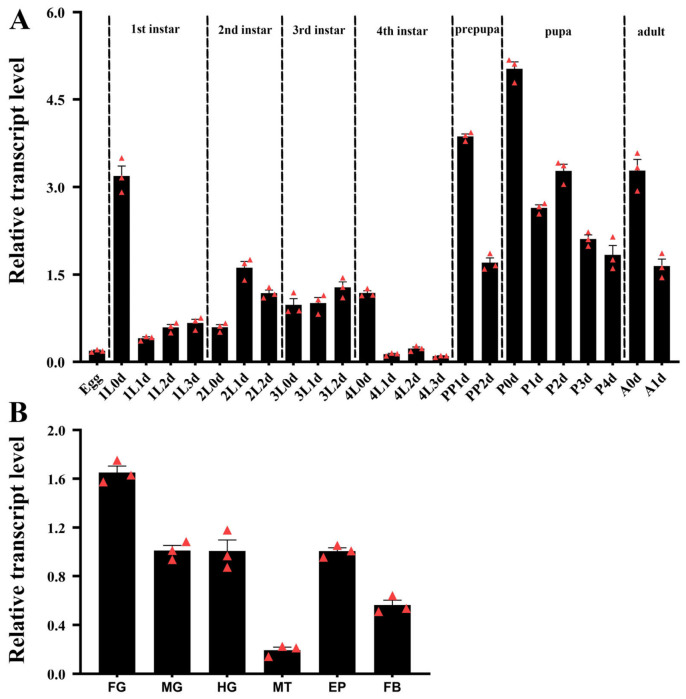
Temporal (**A**) and tissue (**B**) expression of *HvMMP2* in *Henosepilachna vigintioctopunctata*. For temporal expression analysis, RNA templates were derived from eggs (day 3), the larvae from the first through the fourth instars, prepupae, pupae and adults (D0 indicated newly ecdysed larvae or pupae, or newly emerged adults). For tissue expression analysis, the relative transcripts were measured in the foregut (FG), midgut (MG), hindgut (HG), Malpighian tubules (MT), epidermis (EP) and fat body (FB) of the day 2 final instar larvae. For each sample, 3 independent pools of 5–10 individuals were measured in technical triplicate using qRT-PCR. The red triangle indicates biological replicates. The values were calculated using the 2^−ΔΔCT^ method. The relative expression levels are the ratio of the expression levels at different developmental stages to that of the 3L1d larvae, or the ratio of the expression levels in different tissues to that of HG, which are set as 1. The columns represent averages with vertical lines indicating ± SE.

The expression patterns of *HvMMP2* at different developmental stages were analyzed using quantitative real-time PCR (qRT-PCR). The results showed that *HvMMP2* was expressed throughout the entire developmental period from egg to adult. Transcript levels were elevated around the molting periods, with high expression beginning in the prepupal stage, peaking immediately after pupation, and then gradually declining (Figure 3A).

The tissue-specific expression profile of *HvMMP2* was examined in day-2 fourth-instar larvae. The results revealed that *HvMMP2* was broadly expressed in the foregut, midgut, hindgut, Malpighian tubules, epidermis, and fat body. Expression levels were relatively high in the intestinal tract, with the highest expression observed in the foregut, moderate expression in the epidermis and fat body, and low expression in the Malpighian tubules (Figure 3B).

### 3.3. Effect of Knockdown of HvMMP2 in the Fourth Instar Larvae

**Figure 4:**
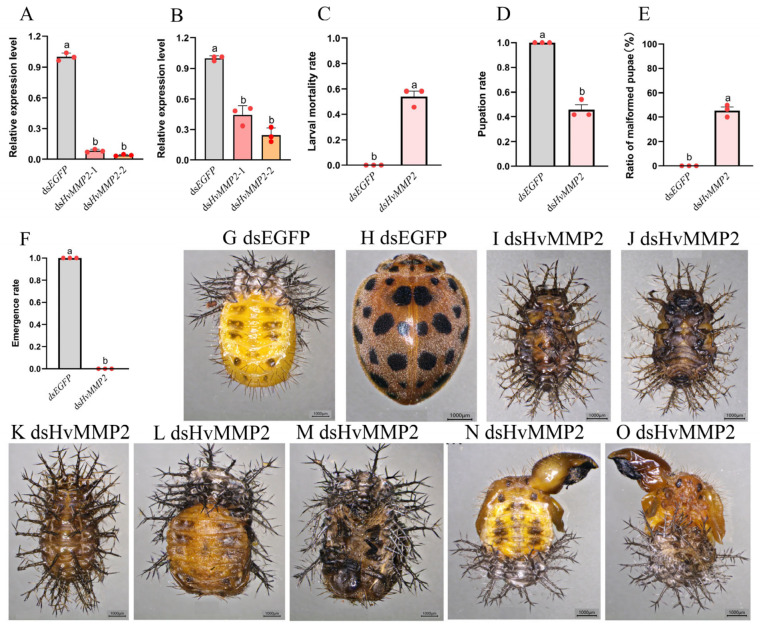
*HvMMP2* silencing impairs pupation and pupal development in fourth instar *Henosepilachna vigintioctopunctata* larvae. Newly molted fourth instar larvae were respectively injected with 400 ng of ds*EGFP* and ds*HvMMP2*. Injected larvae were subsequently transferred to fresh potato leaves for rearing. Expression levels of *HvMMP2* were measured at 24 and 48 h post-injection (**A**,**B**). Relative expression level is defined as the ratio of the expression level of the treated group larvae to that of the ds*EGFP* control group (set as 1). Larval mortality rate (**C**), pupation rate (**D**), ratio of malformed pupae (**E**) and emergence rate (**F**) were recorded. The red circle indicates biological replicates. The columns represent averages with vertical lines indicating ± SE. Different letters denote significant differences at *p* < 0.05. The larvae treated with ds*EGFP* underwent normal pupation (**G**) and eclosion (**H**). The larvae treated with ds*HvMMP2* remained in the prepupal and pupal stages and died (**I**–**O**), and some of the pupae showed abnormal wing development (**N**,**O**).

To investigate the physiological function of *HvMMP2* during larval metamorphosis, ds*HvMMP2* was injected into fourth-instar larvae, and the transcriptional levels of the *HvMMP2* gene were examined 24 and 48 h post-injection. Compared with the ds*EGFP* control group, the transcript level of *HvMMP2* in the ds*HvMMP2*-treated group was significantly reduced (Figure 4A,B). Correspondingly, severe mortality was observed in the ds*HvMMP2*-treated insects. In contrast to the control group, in which 100% of larvae successfully pupated and emerged as adults (Figure 4F–H), ds*HvMMP2*-treated larvae exhibited developmental abnormalities. Approximately 54% of the treated larvae failed to pupate and remained in the prepupal stage, where they melanized and died (Figure 4C,I–K). The remaining 46% of treated larvae successfully pupated (Figure 4D,L–O). Approximately 45% of the pupae showed enlarged and deformed wings (Figure 4E,N,O). All pupae were developmentally arrested, unable to eclose, and eventually melanized and died (Figure 4F).

### 3.4. Effect of Knockdown of HvMMP2 in the Third Larval Instars

**Figure 5:**
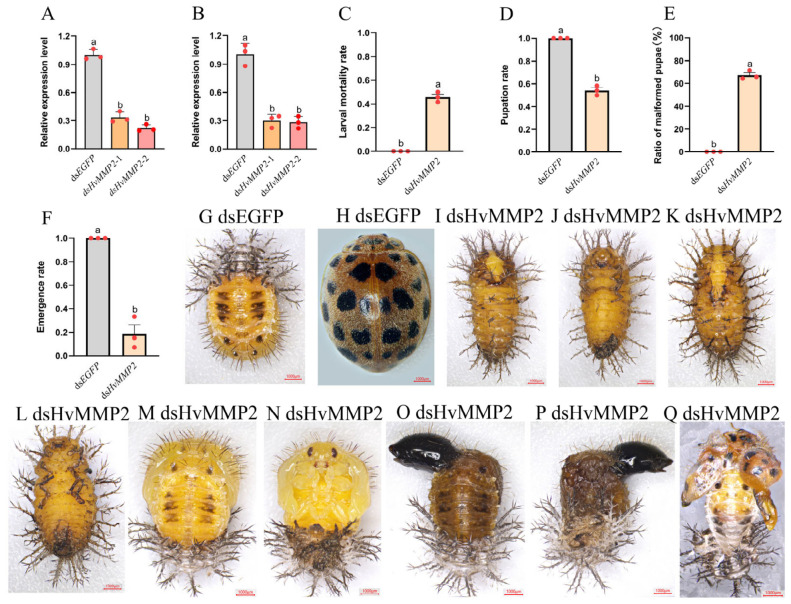
*HvMMP2* knockdown impairs pupation and emergence in third instar *Henosepilachna vigintioctopunctata* larvae. Newly molted third instar larvae were respectively injected with 300 ng of ds*EGFP* and ds*HvMMP2*. Injected larvae were subsequently transferred to fresh potato leaves for rearing. Expression levels of *HvMMP2* were measured at 24 and 48 h post-injection (**A**,**B**). Relative expression level is defined as the ratio of the expression level of the treated group larvae to that of the ds*EGFP* control group (set as 1). Larval mortality rate (**C**), pupation rate (**D**), ratio of malformed pupae (**E**) and emergence rates (**F**) were recorded. The red circle indicates biological replicates. The columns represent averages with vertical lines indicating ± SE. Different letters denote significant differences at *p* < 0.05. The larvae treated with ds*EGFP* underwent normal pupation (**G**) and eclosion (**H**). The larvae treated with ds*HvMMP2* remained in the prepupal and pupal stages and died (**I**–**P**), and some of the pupae showed abnormal wing development (**M**–**P**). Some of the pupae emerged as abnormal adults (**Q**).

To investigate whether *HvMMP2* plays a significant role in the larval molting process, ds*HvMMP2* was injected into third-instar larvae. 24 and 48 h post-injection, the expression of the *HvMMP2* gene in the ds*HvMMP2*-treated group was significantly inhibited compared with the ds*EGFP* control group (Figure 5A,B). However, this marked suppression of *HvMMP2* expression did not affect the molting process from the third to fourth instar, all injected third-instar larvae developed normally into the fourth instar, and only during the transformation from larva to pupa did severe phenotypic defects occur.

Upon reaching the fourth instar, phenotypes similar to those observed following direct fourth-instar interference were observed. In contrast to the ds*EGFP* control group, which underwent complete metamorphosis and successfully emerged as adults (Figure 5F,H), approximately 46% of the treated larvae died during the prepupal stage (Figure 5C,I–L), while the remaining larvae successfully pupated (Figure 5D,M–P). Among these pupae, approximately 64% exhibited enlarged, deformed wings (Figure 5E,M–P). Furthermore, about 19% of the pupae were able to initiate emergence, and they all died during the emergence process (Figure 5F), failing to fully shed the pupal cuticle and displaying wing deformities (Figure 5Q).

### 3.5. HvMMP2 Knockdown Was Associated with Defective Fat Body Remodeling and Abnormal Malpighian Tubule Phenotype

**Figure 6:**
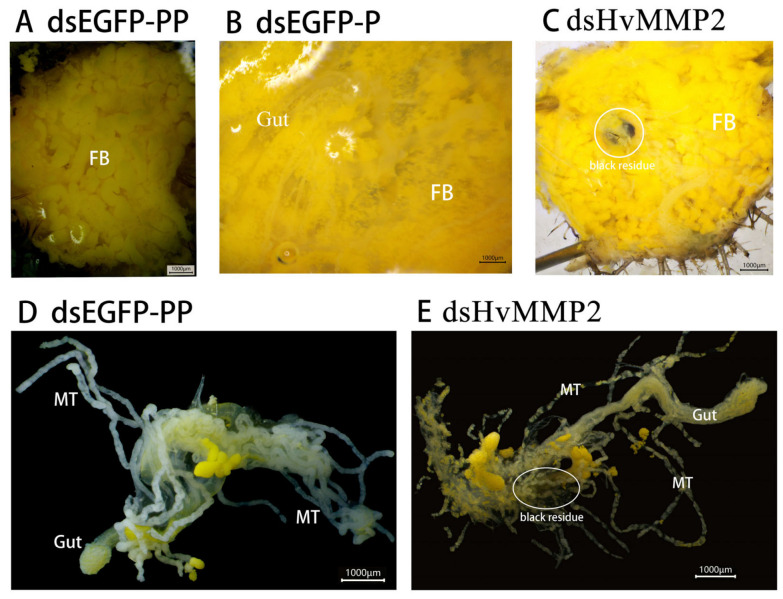
The effect of *HvMMP2* knockdown in *H. vigintioctopunctata* on the fat body and the Malpighian tubules. The fat bodies and intestines of ds*EGFP*-treated larvae at the early pupal stage were presented (**A**,**B**). The fat bodies and intestines of ds*HvMMP2*-treated larvae at the prepupal stage were shown (**C**). The intestines and Malpighian tubules of ds*EGFP*-treated larvae at the prepupal stage were displayed (**D**). The Malpighian tubules and intestines of ds*HvMMP2*-treated larvae at the prepupal stage were shown (**E**).

To investigate the effects of *HvMMP2* knockdown on larvae that became arrested and died at the prepupal stage, microdissection was performed. The results showed that in the control larvae treated with ds*EGFP*, a remodeling of the fat body during the molting process was observed (Figure 6A,B), while the fat bodies of the larvae treated with ds*HvMMP2* showed abnormal morphology, presenting as large masses (Figure 6C). This is consistent with the situation where fat body remodeling has failed. Notably, compared to ds*EGFP* treatment (Figure 6D), the Malpighian tubules of the larvae treated with ds*HvMMP2* were associated with urate crystal accumulation (Figure 6E), suggesting impaired excretory function. Furthermore, ds*EGFP*-treated control larvae exhibited normal intestinal clearance and developed to the pupal stage (Figure 6B). In contrast, ds*HvMMP2* treatment showed incomplete intestinal clearance, with a small amount of black residue remaining in the intestines (Figure 6C,E).

## 4. Discussion

At present, relatively little attention has been given to genes encoding matrix metalloproteinases in insect research. In this study, we identified two transcript variants of the *HvMMP2* gene in *H. vigintioctopunctata*. We found that *HvMMP2* knockdown impaired pupation and adult emergence.

### 4.1. Molecular Characterization and Evolutionary Conservation of HvMMP2

In this study, two transcript variants of *HvMMP2*, namely *HvMMP2x1* and *HvMMP2x2*, were identified from *H. vigintioctopunctata*. Both subtypes encode proteins containing the canonical Peptidase_M10 domain, hemopexin-like repeats, and putative peptidoglycan-binding domains. The presence of the zinc-binding motif (HEXXHXXGXXH) within the Peptidase_M10 domain confirms that *HvMMP2* belongs to the matrix metalloproteinase (MMP) family, which relies on zinc for catalytic activity [34]. Notably, the two transcript variants share identical core regions but differ in their 5′ UTRs and N-terminal amino acid sequences, suggesting potential regulatory or subcellular functional divergence. Phylogenetic analysis revealed that *HvMMP2* clusters tightly with *MMP2* orthologs from *T. castaneum* and *Dendroctonus ponderosae* within Coleoptera, indicating that the function of *HvMMP2* is evolutionarily conserved among beetles. This conserved clustering is consistent with the possibility that *HvMMP2* may play roles similar to its orthologs, such as involvement in ECM remodeling during development and metamorphosis [35].

### 4.2. Spatiotemporal Expression Patterns of HvMMP2 Correlate with Molting and Metamorphosis

Quantitative real-time PCR demonstrated that *HvMMP2* is expressed throughout all developmental stages from egg to adult, with elevated transcript levels around molting periods. The expression peak occurred immediately after pupation, followed by a gradual decline. This is consistent with a role in ECM remodeling during metamorphosis [36]. Similar expression surges around pupation have been observed for *MMP2* in *Aethina tumida* and *B. mori*, where MMP activity is required for larval–pupal transformation [26,37]. Tissue-specific profiling in fourth-instar larvae revealed broad expression in the foregut, midgut, hindgut, Malpighian tubules, epidermis, and fat body, with the highest levels in the foregut. The high expression in the alimentary canal is consistent with the need for extensive gut remodeling during metamorphosis, including programmed cell death of larval epithelial cells and replacement by adult precursors [38]. Moderate expression in the fat body aligns with the roles of MMPs in fat body dissociation [39]. Although *HvMMP2* was found to be expressed at a low level in the Malpighian tubules, based on our RNAi phenotype, the silencing of *HvMMP2* seems to be associated with the accumulation of uric acid in the Malpighian tubules.

### 4.3. Functional Significance of HvMMP2 in Larval Metamorphosis: Tissue Remodeling and Excretion

RNAi-mediated knockdown of *HvMMP2* in fourth-instar larvae caused severe developmental defects, including prepupal arrest with melanization, failed pupation, wing deformities, and complete inability to eclose. These phenotypes are reminiscent of those observed upon MMP inhibition in other holometabolous insects, where MMPs are essential for the breakdown of larval tissues and the formation of adult structures [24,26,40,41]. The abnormal fat body morphology observed in the prepupae treated with ds*HvMMP2*—where fat bodies retained their large, unremodeled morphology—is consistent with the situation where fat body remodeling has failed [42]. The presence of residual black substances in the intestines suggests that the silencing of *HvMMP2* may be associated with the obstruction of the larval intestinal cleaning behavior. One possible explanation is that the intestinal excretion process involves detachment of larval midgut epithelium and its replacement by imaginal cells [24]. Strikingly, the Malpighian tubules of ds*HvMMP2*-treated larvae became slender and accumulated yellow urate crystals. One possible explanation is that *HvMMP2* may regulate the patency or contractility of Malpighian tubules, possibly through ECM turnover around the tubules or via modulation of epithelial integrity. The fact that knockdown of *HvMMP2* in third-instar larvae did not affect molting to the fourth instar, but later induced similar metamorphic defects, indicates that *HvMMP2* is dispensable for larval–larval molting but becomes critically required during the larval–pupal transition. This stage-specific requirement may reflect differences in ECM dynamics and tissue remodeling intensity between larval molts and complete metamorphosis [43,44].

## 5. Conclusions

Collectively, our results support an important role for *HvMMP2* in metamorphic remodeling in *H. vigintioctopunctata* and indicate that this gene may be considered in future studies evaluating RNAi-based control strategies.

## Data Availability

The original contributions presented in this study are included in the article/Supplementary Materials. Further inquiries can be directed to the corresponding authors.

## Supplementary Materials

The following supporting information can be downloaded at: https://www.mdpi.com/article/10.3390/insects17050494/s1, Figure S1: Alignment of nucleic acid sequences of *HvMMP2* isoforms from *Henosepilachna vigintioctopunctata*; Figure S2: Analysis of the statistical results of interfering with ds*HvMMP2-1* and ds*HvMMP2-2* in the fourth and third instar larvae; Table S1: Primers used in RT-PCR, dsRNA synthesis and qPCR.
