# Supplementary material for: RNAi of HvMMP2 Affects Larval-Pupal Transition and Adult Eclosion in the Henosepilachna vigintioctopunctata
Source: Insects. 2026 May 13;17(5):494. doi: 10.3390/insects17050494 (PMC13206845; doi:10.3390/insects17050494)
Supplement: Supplementary file 1 [file insects-17-00494-s001.zip › insects-4262807-supplementary.pdf]

Supplementary data

RNAi of *HvMMP2* Affects Larval-Pupal Transition and Adult Eclosion in the *Henosepilachna vigintioctopunctata*

Jian-Jian Wu*, Meng-Yue Chang, Chen-Yi Wang, Yi-Fan Guo, Kun-peng Cui, Hao Yu*

School of Plant Protection and Environment, Henan Institute of Science and Technology, Exotic Invasive Species Biosecurity Control Innovation Team, Xinxiang, Henan Province 453003, China.

Table S1. Primers used in RT-PCR, dsRNA synthesis and qPCR

Fragment name	Forward primer	Reverse primer
**RT-PCR**		
HvMMP2 X1	TCAGTGTTCGTAGAGGTTTTGTG	GCGTCTCGTGATGATTTTTGT
HvMMP2 X2	CACGGGCGCTATATTTTCT	TCACACCCTGTATGTTCTTCAA
**dsRNA synthesis**		
ds*HvMMP2-1*	ACATTCATTGGGTTTAGCC	TGTTCTATGGGTCCTCGG
ds*HvMMP2-2*	CCACCCAACAGACCTGTACC	CCCAGACCATAGCTCCATCC
ds*egfp*	AAGTTCAGCGTGTCCG	CACCTTGATGCCGTTC
**qPCR**		
qHvMMP2	TATTCCTGTGACTGGCCGTT	TCGTAGTAATCCAGCCCCAC
qHvRPS18	CGCAATCAAAGGTGTTGGAAG	GCCTAGGGTTGGCCATAATAG
qHvRPL13	AGCATCCTTCGCTCGTTTAG	TTCGACAACCTGCCATTAGG

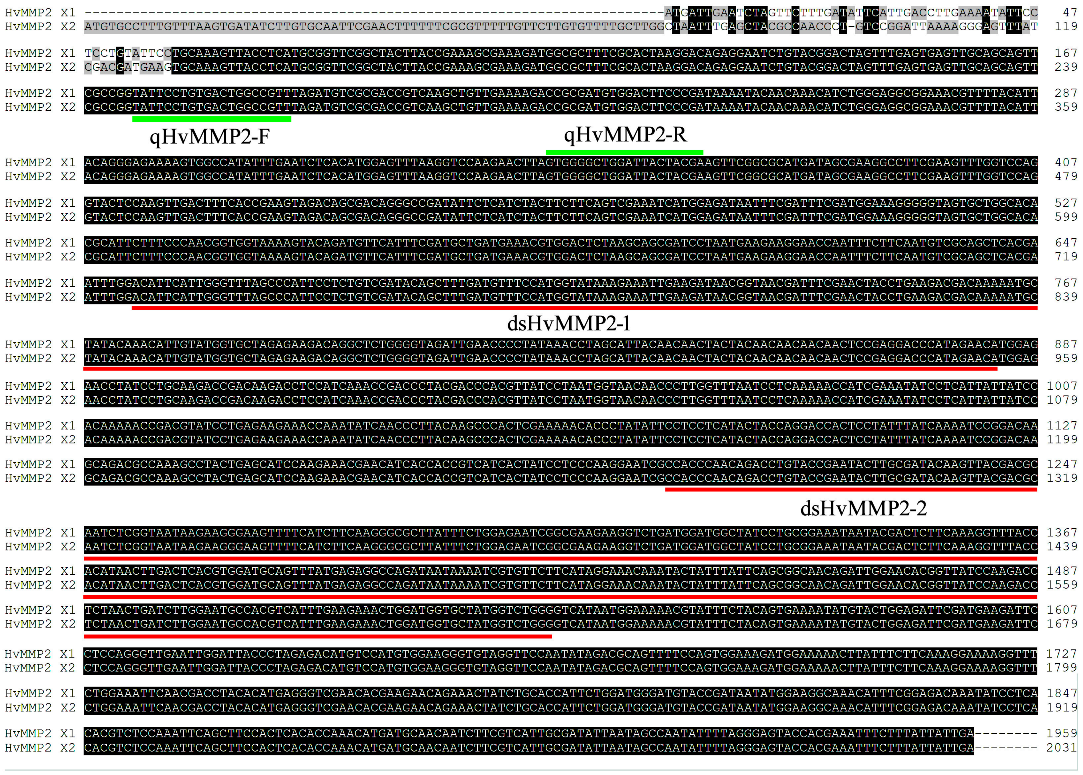

Figure S1. Alignment of nucleic acid sequences of *HvMMP2* transcript variants from *Henosepilachna vigintioctopunctata*. Two *HvMMP2* transcript variants are aligned. *HvMMP2 X1* and *HvMMP2 X2* have different 5’UTR. The sequences for qRT-PCR are marked; and the sequences of ds*HvMMP2* is highlighted.

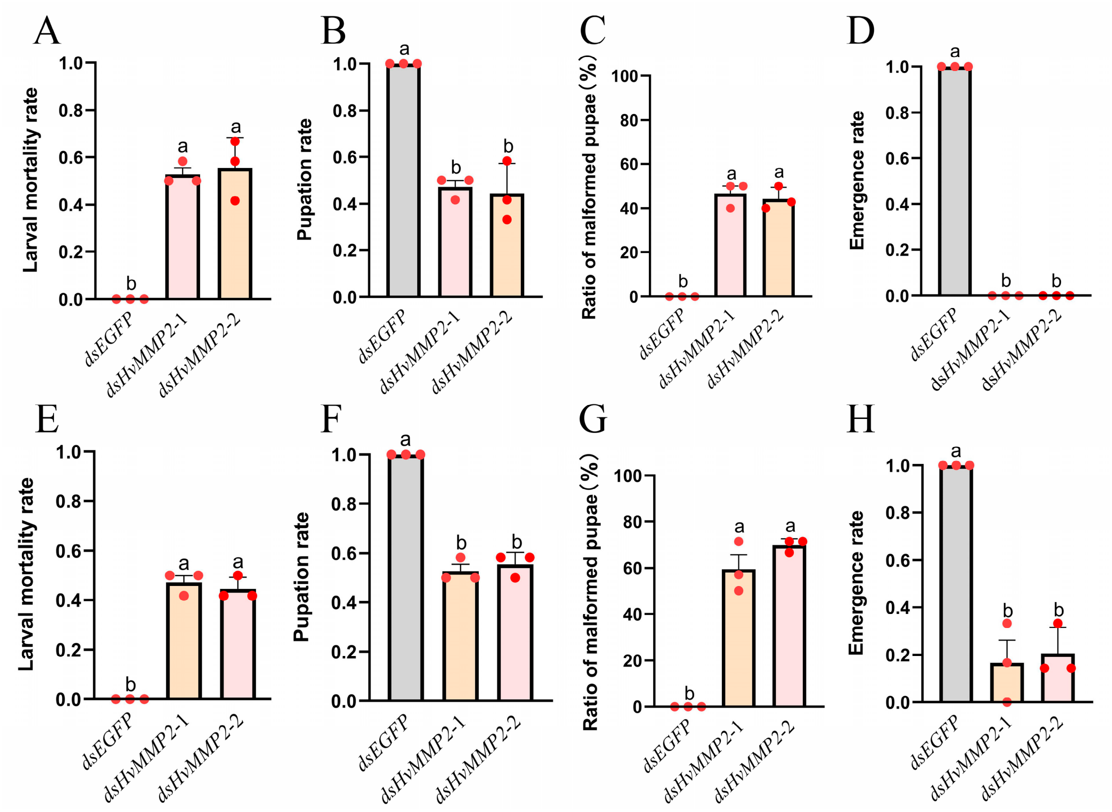

Figure S2. Analysis of the statistical results of interfering with ds*HvMMP2*-1 and ds*HvMMP2*-2 in the fourth and third instar larvae. Larval mortality rate (A), pupation rate (B), ratio of malformed pupae (C) and emergence rate (D) were recorded in the fourth instar larvae. Larval mortality rate (E), pupation rate (F), ratio of malformed pupae (G) and emergence rate (H) were recorded in the third instar larvae. The columns represent averages with vertical lines indicating ± SE. Different letters denote significant differences at $p < 0.05$.
